# Examining inequalities in uptake of maternal health care and choice of provider in underserved urban areas of Mumbai, India: a mixed methods study

**DOI:** 10.1186/s12884-015-0661-6

**Published:** 2015-09-28

**Authors:** Glyn Alcock, Sushmita Das, Neena Shah More, Ketaki Hate, Sharda More, Shanti Pantvaidya, David Osrin, Tanja AJ Houweling

**Affiliations:** Institute for Global Health, University College London, 30 Guilford Street, London, WC1N 1EH UK; Society for Nutrition, Education and Health Action (SNEHA), 310, 3rd Floor, Urban Health Center, 60 Feet Road, Dharavi, Mumbai 400 017 India; Department of Public Health, Erasmus MC University Medical Center Rotterdam, P.O. Box 2040, 3000 CA Rotterdam, The Netherlands

**Keywords:** Maternal health, Health inequalities, Health care utilisation, Determinants of care, Urban slums, India

## Abstract

**Background:**

Discussions of maternity care in developing countries tend to emphasise service uptake and overlook choice of provider. Understanding how families choose among health providers is essential to addressing inequitable access to care. Our objectives were to quantify the determinants and choice of maternity care provider in Mumbai’s informal urban settlements, and to explore the reasons underlying their choices.

**Methods:**

The study was conducted in informal urban communities in eastern Mumbai. We developed regression models using data from a census of married women aged 15–49 to test for associations between maternal characteristics and uptake of care and choice of provider. We then conducted seven focus group discussions and 16 in-depth interviews with purposively selected participants, and used grounded theory methods to examine the reasons for their choices.

**Results:**

Three thousand eight hundred forty-eight women who had given birth in the preceding 2 years were interviewed in the census. The odds of institutional prenatal and delivery care increased with education, economic status, and duration of residence in Mumbai, and decreased with parity. Tertiary public hospitals were the commonest site of care, but there was a preference for private hospitals with increasing socio-economic status. Women were more likely to use tertiary public hospitals for delivery if they had fewer children and were Hindu. The odds of delivery in the private sector increased with maternal education, wealth, age, recent arrival in Mumbai, and Muslim faith. Four processes were identified in choosing a health care provider: exploring the options, defining a sphere of access, negotiating autonomy, and protective reasoning. Women seeking a positive health experience and outcome adopted strategies to select the best or most suitable, accessible provider.

**Conclusions:**

In Mumbai’s informal settlements, institutional maternity care is the norm, except among recent migrants. Poor perceptions of primary public health facilities often cause residents to bypass them in favour of tertiary hospitals or private sector facilities. Families follow a complex selection process, mediated by their ability to mobilise economic and social resources, and a concern for positive experiences of health care and outcomes. Health managers must ensure quality services, a functioning regulatory mechanism, and monitoring of provider behaviour.

## Background

Poor coverage and low uptake of skilled maternity care are major contributors to maternal morbidity and mortality. India alone accounts for 17 % of the 289 000 annual global pregnancy-related deaths [1]. Safe motherhood requires adequate distribution of health services, access to emergency obstetric care, and skilled birth attendance [2].

Individual, household, community, and health system factors affect access to and utilisation of health care. At the individual and household levels, economic status is a key determinant. Analysis of Demographic and Health Survey (DHS) data from 45 developing countries has shown that wealthier women are much more likely than poorer women to have prenatal care and to deliver with a skilled attendant [3]. Other country-level studies support this trend. In Nigeria, women in the highest household wealth quintile are at least seven times more likely to deliver in a health facility than women in the lowest [4]. In Cambodia, the wealthiest women are almost 12 times more likely to do so [5].

Other determinants include maternal age, education, and parity. For example, younger, less educated women from lower socioeconomic groups in Brazil make inadequate use of prenatal care services [6]. In Bangladesh, skilled maternity care among married adolescents is associated with higher education and wealth index, urban residence, and lower birth order [7]. In India, women in northern states and rural areas use maternal health care less than others; barriers include low household economic status, caste, maternal and paternal education, higher birth order, Muslim faith, and less exposure to mass media [8, 9].

Some research suggests that women’s autonomy effects maternal care-seeking [10–12]. In Ethiopia, women who were ultimately responsible for decisions about birthplace were almost four times more likely to deliver at a health facility than those who were not [13]. In Tajikistan, women with financial decision-making power were more likely to attend at least one prenatal consultation (although less likely to attend four or more), deliver with a skilled provider, and seek institutional delivery care. However, associations are contextual; autonomy might be a weak predictor of care uptake in general [14], but more strongly associated with choosing private over public sector care [15].

Studies in diverse settings have shown associations between urban location and institutional delivery [5, 7, 16]. Urban residents benefit from a concentration of health infrastructure and proximity of services. However, population growth creates greater demand for health services. When these services are unevenly distributed, access becomes unequal. These inequalities adversely affect disadvantaged groups in underserved neighbourhoods [17]. Our previous research has shown a positive association between higher socioeconomic status and the use of private prenatal and delivery care by women from informal urban settlements (slums) in Mumbai [18].

India is the world’s fastest urbanising country; currently, 410 million Indians (one-third of the total population) live in urban areas. Mumbai, the country’s second largest city, has more than 16 million inhabitants [19], more than 40 % of whom live in slum areas [20]. The health care sector is characterised by a co-existence of medical systems and public and private providers. Public sector infrastructure includes teaching hospitals, specialist hospitals, general hospitals, maternity hospitals, and community-level health posts and dispensaries [21]. The private sector includes super-speciality hospitals, medium-sized facilities that provide both outpatient and inpatient care, and a substantial number of smaller practices that offer limited services. Most urban health care across socioeconomic groups, including the disadvantaged, is privately provided. The sector is virtually unregulated and many practitioners are underqualified or lack formal training [22, 23].

Because institutional prenatal and delivery services are often underutilised, discussions of maternity care in low- and middle-income countries have emphasised uptake of services, followed by a consideration of quality. While some research has documented the utilisation of public and private sector services [5, 24, 25], choice of specific types of facility within each sector has largely been ignored. Understanding how families in underserved urban communities choose among health providers is essential. Although the public sector is an important source of health care for the urban poor, private practitioners dominate in many low-income communities. Examining health care-seeking behaviours in these communities is key to developing effective strategies that address inequalities, improve access, and help protect the poor against unaffordable health costs [26].

Our objectives were to quantify the pattern, determinants, and choice of maternity care provider at the health facility level in the public and private sectors in Mumbai’s informal urban settlements, and to explore the reasons underlying these choices. We were interested in examining two aspects of choice that have appeared rarely in discussions: private sector maternity care for poor people whose substantial use of it has gone largely unnoticed, and the ways in which they decide which providers they will use. Our broad hypothesis was that the likelihood of institutional prenatal care, delivery, and private health care would all increase as maternal education, duration of residency, and economic status increased.

## Methods

### Study setting

The study was conducted in informal settlements in two eastern municipal wards in Mumbai (M East and L). Both rank lowest on the UN Human Development Index for the city with a comparatively high concentration of slum residency (78 and 85 % respectively), higher infant mortality, lower life expectancy, and lower female literacy and employment. The majority of residents are of Muslim faith [27]. The two wards were included in a cluster randomised controlled trial of community resource centres. Centres served as a base for the collection and dissemination of health information, home visits, care for malnourished children, referral of individuals and families to appropriate services, meetings of community members and providers, and events and campaigns on health issues [28]. Trial areas comprised 40 informal settlements, each of approximately 600 households, and covered a population of ~120 000.

### Study design, participants, and tools

We used a sequential mixed-methods design [29]. First, we analysed data from a baseline census to describe determinants of maternity care, then used grounded theory methods to examine women’s choice and utilisation of provider. We used this approach in order to (1) describe the quantitative patterns and determinants of maternity care utilisation, (2) from the quantitative results, purposively select individual women from social, economic, and demographic characteristics and choice of health care provider, (3) explore possible relationships between the observed quantitative patterns and determinants of care, and women’s narratives of care-seeking, and (4) triangulate quantitative and qualitative data.

The research team comprised a principal investigator (TH), a senior data manager (SD), a senior researcher (DO), an experienced male qualitative researcher (GA), two female junior qualitative researchers (KH and SM), SNEHA’s Executive Director of Programs (SP) and the Program Director for the resource centre trial (NSM).

We used two datasets in the study: the trial baseline census for the quantitative analysis and the intervention database to identify participants for qualitative interview. Census respondents were all residents of trial areas and were married women in the 15–49 age group. The actual ages of respondents included in the census ranged from 17–49. The intervention database allowed us to purposively sample individual women based on their care-seeking behaviour and because we did not expect the trial to impact choice of provider. Selection criteria for qualitative interview included married women aged 18 and over who were currently pregnant or had given birth (at home or in a health facility) in the previous two years.

#### Data collection

Quantitative data were collected in a baseline census over 18 months from September 2011 to March 2013. All respondents gave signed consent prior to interview. Interviewers took household GPS coordinates and enumerated household members, their ages, schooling and livelihoods. The interview covered duration of residence, assets and amenities, housing fabric and faith. Women provided brief maternity histories and information on family planning.

Data were collected on smartphones running Open Data Kit (www.opendatakit.org), which included inbuilt skips and validation constraints. After checks for completeness, data were uploaded to a secure database in ODK Aggregate. They were cleaned and analysed in Stata 12 (StataCorp, College Station, Tx: www.stata.com).

We used semi-structured topic guides for qualitative data collection, including sections on the respondent’s background (e.g. place of origin, family structure), experiences of pregnancy and childbirth, maternity care, and choice of provider. Women were explained the purpose of the study and assured of confidentiality before giving verbal consent to participate. KH and SM conducted seven focus groups (alternating between moderating and note-taking) with married women (average, eight per group), 16 in-depth interviews, one group discussion with five SNEHA Community Organisers, and an interview with the mother-in-law of two respondents. In total, 78 women from nine clusters participated. Focus groups took place at the nearest nongovernment outreach centre and most interviews in the participant’s home. They were conducted in Hindi or Marathi and lasted from 30 min to over an hour. We stopped data collection when we felt concepts and themes were sufficiently developed.

Focus groups and interviews were digitally recorded and transferred to two password-protected computers. The interviewers anonymised and transcribed their own interviews verbatim and translated them into English for dissemination among the research team. Translated transcripts were randomly selected and cross-checked for accuracy.

### Analysis

#### Quantitative analysis

##### Dependent variables

We were interested in examining uptake of prenatal and institutional delivery care, whether it was in the public or private sector, and whether women’s choices favoured tertiary public hospitals. We defined prenatal care as attendance for at least three check-ups (the locally recommended minimum). Public sector facilities providing prenatal care included municipal health posts, urban health centres, maternity homes, general hospitals, and tertiary hospitals. We included established, large state government hospitals in the latter group as they provide free or low-cost services. Delivery was possible at all these types of facility except for health posts. Private sector facilities included single-handed practices without inpatient services, small maternity homes and inpatient centres, and larger hospitals. Delivery was possible at all but single-handed facilities without beds.

##### Independent variables

We chose variables purposively from the available dataset, to reflect socio-economic position (household asset index, maternal schooling), demography (maternal age, parity), establishment and familiarity with healthcare options (duration of residence), and socio-cultural milieu (faith). Maternal schooling was described in an ordered categorical variable as none, primary, secondary, or higher than secondary. Socio-economic position was described by quintiles of an asset index developed from standardized weights of the first component of a principal components analysis [30, 31]. Assets included home ownership, possession of a ration card, robust housing fabric, private water supply, private toilet, finished floor, and possession of a mattress, pressure cooker, gas cylinder, stove, bed, table, clock, mixer, telephone, refrigerator, or television. Duration of residence was a continuous variable describing the number of years the woman had been living in Mumbai. A continuous variable describing parity included the index pregnancy in the preceding two years. Faith was categorized as a binary variable describing Muslim or other faith.

##### Statistical analysis

The analyses included women who had reported a birth in the 2 years preceding the census. We tabulated frequencies and percentages of attendance for prenatal care, its location in the private or public sector, and the use of tertiary hospitals and smaller public sector institutions, against the chosen independent variables. We did the same for institutional delivery.

For each combination of dependent and independent variables, we developed a univariable logistic regression model with a random effect for cluster. For prenatal care, whether the woman had 3 or more visits (denominator: all women who had had a pregnancy in the preceding 2 years), whether the prenatal care was in the public rather than the private sector (denominator: women who had made more than 3 prenatal care visits), and whether it was in a large public hospital rather than a smaller one (denominator: women who had made more than 3 prenatal visits in the public sector). For delivery, whether institutional or at home (denominator: women had had delivered in the preceding 2 years), whether it was in the public rather than the private sector (denominator: women who had had an institutional delivery), and whether it was in a large public hospital rather than a smaller one (denominator: women who had delivered in the public sector).

For each outcome, we created a single multivariable logistic regression model with random effect for cluster. All models included adjustment covariates selected as markers of socio-economic position, demography, establishment and familiarity with healthcare options, and socio-cultural milieu. Age and parity were both included in the models since the Stata *collin* package did not suggest collinearity. All models satisfied quadrature parameters.

#### Qualitative analysis

We used grounded theory (GT) methods. GT is an inductive research methodology to generate theory through the development of conceptual categories that are *grounded* in systematically collected and analysed data [32, 33]. We coded the English transcripts in NVivo version 10 (QSR International: http://www.qsrinternational.com).We began by open coding transcripts individually and analysed them collectively to identify and explore descriptive and higher-level conceptual categories. We tested emerging categories and interpretation through constant comparison and presentations to colleagues.

Ethical approval for the study was granted by the UCL Ethics Committee and the Multi-institutional Ethics Committee of the Anusandhan Trust in Mumbai.

## Results

### Quantitative

Data for the study were provided by 3848 women who had delivered a baby in the preceding two years. Table 1 presents information on these women. Just over half had some secondary education and more than half said that they had lived in Mumbai for at least 10 years. Most (74 %) were in the age group 20–29 years and 56 % had one or two children. Most were Muslim (83 %).

**Table 1:** Characteristics of 3848 women respondents in 40 informal settlement areas in Mumbai who had delivered in the two years preceding the census

	Respondents	(%)
Maternal education		
None or informal	1170	(30)
Primary	236	(6)
Secondary	2144	(56)
Higher	297	(8)
Missing	1	(<1)
Household asset quintile		
Quintile 1	771	(20)
Quintile 2	769	(20)
Quintile 3	785	(20)
Quintile 4	758	(20)
Quintile 5	765	(20)
Duration of residency in Mumbai		
Less than 1 year	260	(7)
1–4 years	867	(22)
5–9 years	561	(15)
10 years or more	2160	(56)
Age		
Under 20	139	(3)
20–29	2841	(74)
30–39	804	(21)
40–49	64	(2)
Parity, including index delivery		
1	1168	(30)
2	1009	(26)
3	765	(20)
4	413	(11)
5 or more	493	(13)
Religion		
Muslim	3184	(83)
Hindu	651	(17)
Other	13	(<1)
All	3848	(100)

**Fig. 1:**
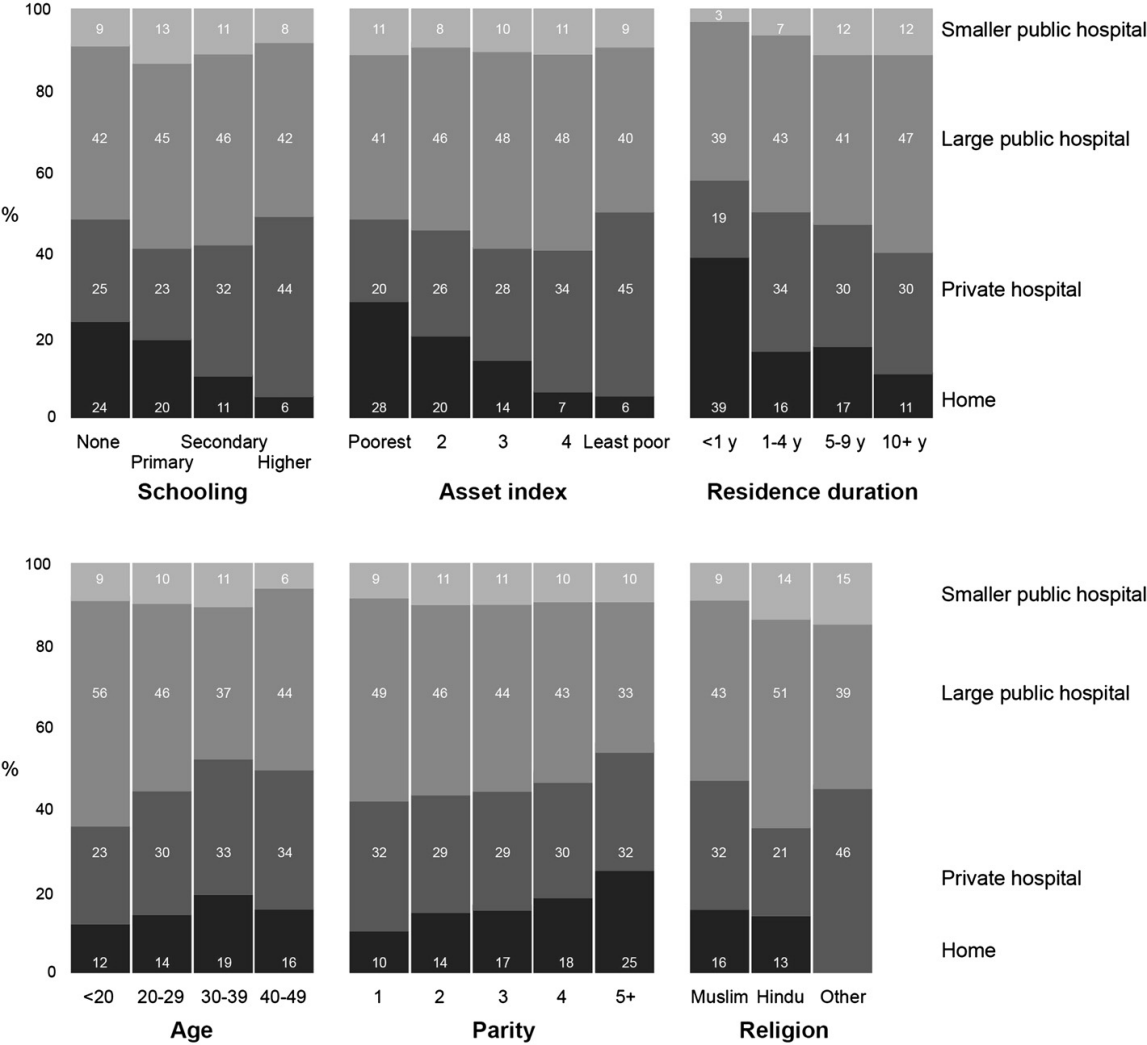
Delivery care site, by maternal characteristics, for 3820 deliveries in the two years preceding the study. Note: Information missing for 28 women

Table 2 summarizes choice of prenatal care provider and Fig. 1 delivery care for all women who had delivered in the preceding two years for the whole sample and by socio-economic and socio-demographic characteristics. Overall, institutional maternity care-seeking was high: 94 % made three or more prenatal visits and 85 % had a facility delivery. Uptake of prenatal care and institutional delivery care was lower for women who never went to school, were poorer, and who had recently arrived in Mumbai. Note that in our sample the wealthiest were simply the least poor quintile group in a vulnerable urban slum population. Uptake of prenatal and delivery care was also lower for older women with more children. Within the public sector, there was a preference for tertiary (municipal or state) hospitals across all socio-economic positions, although this fell with increasing parity. Preference for private hospitals, for both prenatal and delivery care, increased with household economic status. A greater proportion of Muslim women went to private hospitals for prenatal care and delivery (33 and 32 %, respectively) than Hindu women (18 and 21 %, respectively). The longer women had lived in Mumbai, the higher the proportion who went to tertiary public hospitals, especially for prenatal care.

**Table 2:** Prenatal care site, by maternal characteristics, for 3819 deliveries in the two years preceding the census

				Prenatal care in public sector	
	Total	<3 prenatal care visits	Prenatal care in private sector	Prenatal care at tertiary public hospital	Prenatal care at smaller public facility
	*n* (%)	*n* (%)	*n* (%)	*n* (%)	*n* (%)
All	3819 (100)	242 (6)	1160 (31)	1880 (49)	537 (14)
Maternal education					
None or informal	1158 (100)	137 (12)	300 (26)	562 (49)	159 (14)
Primary	234 (100)	14 (6)	54 (23)	124 (53)	42 (18)
Secondary	2130 (100)	89 (4)	672 (32)	1065 (50)	304 (14)
Higher	297 (100)	2 (1)	134 (45)	129 (43)	32 (11)
Household asset quintile					
Quintile 1	765 (100)	114 (15)	152 (20)	377 (49)	122 (16)
Quintile 2	764 (100)	60 (8)	199 (26)	407 (53)	98 (13)
Quintile 3	772 (100)	37 (5)	225 (29)	395 (51)	115 (15)
Quintile 4	755 (100)	19 (3)	234 (31)	393 (52)	109 (14)
Quintile 5	763 (100)	12 (2)	350 (46)	308 (40)	93 (12)
Duration of residency in Mumbai					
Less than 1 year	258 (100)	61 (24)	63 (24)	115 (45)	19 (7)
1–4 years	864 (100)	67 (8)	296 (34)	399 (46)	102 (12)
5–9 years	557 (100)	35 (6)	173 (31)	262 (47)	87(16)
10 years or more	2140 (100)	79 (4)	628 (29)	1104 (52)	329 (15)
Age					
Under 20	139 (100)	4 (3)	31 (22)	86 (62)	18 (13)
20–29	2823 (100)	166 (6)	846 (30)	1427 (50)	384 (14)
30–39	795 (100)	67 (8)	263 (33)	338 (43)	127 (16)
40–49	62 (100)	5 (8)	20 (32)	29 (47)	8 (13)
Parity					
1	1163 (100)	51 (4)	373 (32)	597 (51)	142 (12)
2	1007 (100)	49 (5)	297 (29)	518 (52)	143 (14)
3	758 (100)	53 (7)	217 (29)	372 (49)	116 (15)
4	405 (100)	26 (7)	123 (30)	195 (48)	61 (15)
5 or more	486 (100)	63 (13)	150 (31)	198 (41)	75 (15)
Religion					
Muslim	3161 (100)	198 (6)	1041 (33)	1523 (48)	399 (13)
Hindu	645 (100)	44 (7)	114 (18)	352 (54)	135 (21)
Other	13 (100)	-	5 (38)	5 (38)	3 (23)

Note: Information missing for 29 women

Table 3 summarizes the findings of univariable and multivariable models. The odds of prenatal care increased with education, economic status in terms of household asset quintile, and duration of stay in Mumbai, and decreased with parity. Similar associations were found for institutional delivery. Of those who opted for institutional care, women were more likely to have prenatal care and delivery in the private than the public sector if they were more educated, wealthier, had lived in Mumbai for a shorter time, were older, or were Muslim. Within the public sector, women were more likely to use smaller institutions for prenatal care if they had more children, and for delivery if they had lived in Mumbai longer and had more children.

**Table 3:** Odds ratios for uptake of prenatal care and institutional delivery, care in the public sector, and care at tertiary public hospitals, in the two years preceding the study, by maternal characteristics

	Prenatal care		Delivery care	
	OR (95 % CI)	aOR (95 % CI)	OR (95 % CI)	aOR (95 % CI)
	3 or more prenatal care visits *(reference: <3 visits)*		Institutional delivery *(reference: home delivery)*	
Maternal schooling (y)	1.17 (1.13, 1.22)	1.08 (1.04, 1.13)	1.13 (1.10, 1.16)	1.07 (1.04, 1.10)
Household asset quintile	2.46 (2.08, 2.90)	1.92 (1.59, 2.32)	1.95 (1.75, 2.18)	1.62 (1.43, 1.83)
Duration of residency (y)	1.06 (1.04, 1.08)	1.05 (1.03, 1.07)	1.04 (1.03, 1.05)	1.03 (1.02, 1.04)
Age (y)	0.96 (0.93, 0.98)	1.01 (0.97, 1.05)	0.96 (0.95, 0.98)	1.01 (0.98, 1.03)
Parity	0.81 (0.76, 0.86)	0.79 (0.72, 0.88)	0.84 (0.80, 0.88)	0.82 (0.76, 0.89)
Muslim faith	1.06 (0.72, 1.56)	1.11 (0.74, 1.66)	0.87 (0.65, 1.15)	0.90 (0.67, 1.21)
	Prenatal care in public sector *(reference: private sector)*		Delivery in public sector (*reference: private sector)*	
Maternal schooling (y)	0.96 (0.94, 0.97)	0 .96 (0.94, 0.98)	0.96 (0.94, 0.98)	0.96 (0.94, 0.98)
Household asset quintile	0.72 (0.66, 0.79)	0.70 (0.63, 0.77)	0.73 (0.67, 0.80)	0.71 (0.64, 0.78)
Duration of residency (y)	1.01 (1.00, 1.02)	1.02 (1.01, 1.03)	1.01 (1.00, 1.01)	1.02 (1.01, 1.03)
Age (y)	0.98 (0.96, 0.99)	0.94 (0.92, 0.96)	0.97 (0.96, 0.99)	0.96 (0.93, 0.98)
Parity	0.99 (0.95, 1.04)	1.04 (0.97, 1.11)	0.96 (0.91, 1.00)	0.98 (0.92, 1.05)
Muslim faith	0.58 (0.45, 0.75)	0.51 (0.39, 0.67)	0.63 (0.49, 0.81)	0.58 (0.45, 0.75)
	Prenatal care at tertiary public hospital *(reference: other public facility)*		Delivery at tertiary public hospital *(reference: other public facility)*	
Maternal schooling (y)	1.02 (0.99, 1.05)	1.01 (0.98, 1.04)	1.01 (0.98, 1.04)	1.01 (0.97, 1.04)
Household asset quintile	1.02 (0.91, 1.16)	1.03 (0.90, 1.19)	1.03 (0.90, 1.18)	1.07 (0.92, 1.26)
Duration of residency (y)	0.98 (0.98, 0.99)	0.99 (0.98, 1.00)	0.98 (0.97, 0.99)	0.98 (0.97, 0.99)
Age (y)	0.97 (0.95, 0.99)	1.00 (0.97, 1.03)	0.98 (0.96, 1.01)	1.03 (0.99, 1.06)
Parity	0.88 (0.83, 0.94)	0.87 (0.80, 0.95)	0.88 (0.82, 0.95)	0.84 (0.76, 0.93)
Muslim faith	1.15 (0.87, 1.54)	1.30 (0.96, 1.76)	1.04 (0.74, 1.45)	1.20 (0.84, 1.70)

*OR* odds ratio from univariable logistic regression model with random effect for cluster, *aOR* adjusted odds ratio from multivariable logistic regression model, including the other independent variables and random effect for cluster, *CI* confidence interval, *y* years

### Qualitative findings

We identified four conceptual processes in choosing a maternity care provider: exploring the options, defining a sphere of access, negotiating autonomy, and protective reasoning. Health care decisions took place in a context of uncertainty about provider competence, quality of services, and costs and outcomes of care. Strategies aimed at selecting the best or most suitable, accessible health care provider were used, with the underlying goals of ensuring positive health outcomes and avoiding poor quality care and experiences.

#### Exploring the options

Women sought various types of information from relatives, friends and neighbours to identify suitable (and unsuitable) health care providers among unfamiliar alternatives. Suitability was categorised in terms of convenience, affordability, quality, and expected health outcomes. The extent to which women explored options depended on their existing knowledge and experience of maternity and health care. For example, primigravid women knew little about pregnancy and childbirth, and recent migrants had limited knowledge of health facilities and the quality of services: “We were new here … we did not know anything about this place, which hospital is good.” Enquiring with familiar or trusted people provided information about appropriate options.

Then she told me … “Go here [to this private hospital]. The thing is that less [money] will be required here. Today is Sunday. If you register today, you will have to pay 50 Rupees. If you go after today or any other day then they will take 250 Rupees, or whatever it is. And, sister, you don’t have the money.”

(Muslim, delivered at a private hospital)

Advice and recommendations influenced choices so that, “if she knows that this hospital is nice, then she will advise me to go there, and I’ll go.” Similarly, endorsement of a provider, such as, “My brother’s wife delivered a baby boy at this hospital and everything went well”, gave reassurances about a provider’s competence. Additional information, such as provider practices and fees, enabled families to incorporate dimensions of acceptability and affordability into their decisions.

#### Defining a sphere of access

Economic and social status pervaded health care decisions. A convergence of household financial capacity and *aukaad* (social status) and the cost of care across sectors acted as a reference point from which families defined their sphere of access to care. Although the private sector was lauded because “the facilities are good, they give proper medicines and care”, utilisation was contingent on the sphere of access. Since women from lower economic groups had a narrower sphere of access, their choices were usually limited to municipal facilities or inexpensive private providers.

We don’t have the status to pay for private. Out of helplessness, one goes more to government.

(Focus group participant, home birth and public hospital delivery)

Because of all these [financial] problems, we have registered in a municipal hospital.

(Muslim, registered pregnancy at municipal peripheral hospital)

Some families used strategies to access the private sector, even temporarily. These included pooling or borrowing money, or switching sectors if private care became unaffordable.

If the time comes for a caesarean, because of money issues it has to be done in a municipal hospital … because the operation isn’t cheap, [privately] it costs 20–25 thousand Rupees. If we get it done in the municipality the expense will be less and [the money] can be used for food. So, we will have to think if the time comes for an operation.

(Muslim, registered pregnancy at a private clinic)

Besides the financial vulnerability families faced in balancing health care decisions against household sustenance, the excerpt illustrates the provisional and situational nature of choice throughout the care trajectory: they were made according to current financial capacity and re-evaluated for each care-seeking episode or in the event of new financial or medical circumstances.

#### Negotiating autonomy

Seeking care involved mobilising financial and social resources, and decisions often had consequences for household functioning. Choosing an initial or different provider often depended on the women’s ability to negotiate their economic and social conditions. Depending on the location and type of provider, besides the direct and indirect costs of care, relatives or friends were routinely required to accompany women to consultations or help out at home. Since institutional care involved absence from domestic work, potential disruption to the family also had to be considered.

If they had sent me to hospital F my husband would have spent all day travelling to and from the hospital. Not only would he lose an entire day at work, but even my children would be neglected. There would have been no one to look after me regularly there at hospital F. So, I chose this [private nursing home].

(Hindu, delivered at a private nursing home)

Health care choices, therefore, were considered within the economic condition of the household and the women’s social position or had to be modifiable through negotiation. Those unable to mobilise sufficient resources to access a preferred provider compromised: “The hospital is near … we can go and come back quickly and do our household chores.” Women with better access to funds or greater social support and autonomy were able to select a preferred provider. One respondent, for example, chose her prenatal and delivery care with a well-known private doctor in a neighbouring district 12 h away by train.

#### Protective reasoning

Uncertainty about maternal health and health care caused fear and anxiety, and pregnancy and childbirth were considered risky events. Care seeking often emphasised safety and positive health outcomes.

The delivery should be safe and successful. A woman is standing near the mouth of death [during pregnancy] … *Allah tallah* (by God’s blessings), hopefully everything should be fine.

(Mother-in-Law of a woman who delivered at a private facility)

Crucial to health care decisions and choice of provider were a “safe and successful” birth, protection against risk, and avoidance of negative experiences. Consultation with a trusted or reputable provider reassured families that complications would be avoided or resolved and, therefore, institutional care was the norm. Of 13 respondents who had delivered at home, only one had been planned. Other reasons included being unable to go to the hospital alone, being turned away from a health facility either because the due date was later or the woman was not registered. In some cases, hospital staff had been unavailable or unwilling to attend to them at that time.

From their interactions with health care providers and services, women reconceptualised care, which informed subsequent care-seeking preferences and behaviour: positive experiences (e.g. attentive staff and competent doctors who “give good medicines,” and accessible, well-equipped hospitals in convenient locations) produced *attractive* responses, including repeating care at a previously-utilised facility. Negative experiences and perceptions (e.g. abusive provider behaviour, long queues and lengthy administrative procedures, or poorly-equipped hospitals) provoked *aversive* reactions and avoidance strategies.

I won’t go to hospital F (municipal tertiary) … because hospital F is very bad. If someone goes there, she doesn’t return alive.

(Hindu, four deliveries in municipal peripheral hospitals)

Avoidance strategies usually involved discontinuing care with a provider and strategising to seek alternative care. One respondent ceased care a public hospital because of exasperation with being “made to run around” while attempting to register for delivery. Other families sought loans to switch from public to private sector care. In one case, fear of being made to undergo caesarean section led one respondent to abandon all institutional care in favour of home birth.

## Discussion

Our study shows that institutional delivery is the norm in Mumbai’s informal settlements. However, poorer and less educated women, and recent migrants were less likely to receive professional prenatal and delivery care. Tertiary public hospitals were a common source of maternity care across all socioeconomic groups. Private hospitals were popular with wealthier, more educated women.

We identified four conceptual processes central to choosing a health care provider: exploring the options, defining a sphere of access, negotiating autonomy, and protective reasoning. The overall aim was the selection of a suitable or best-option provider. Evidence of quality and positive outcomes encouraged women to seek care with certain providers while others were avoided or abandoned. Heath care decisions and provider choice were mediated by household socio-economic status, the cost of care, and the ability of women to negotiate their social and economic environment.

The dominance of tertiary public hospitals as a preferred site of maternity care across socioeconomic groups is a problem for the equitable delivery of health services to underserved areas: despite being located in proximity to poor neighbourhoods, poor perceptions of quality, limited services and understaffing in primary public health facilities often cause residents to bypass them in favour of tertiary hospitals. To most women, large hospitals symbolised comprehensive, integrated care, sophisticated equipment and technology, expertise and specialisation, where complications could be treated in one place. This made them attractive and convenient. At the same time, this preference exacerbates problems of overcrowding, longer waiting times, shorter consultations in tertiary facilities and loss of wages, dissuading some educated and wealthier people from utilising public sector health care [34].

Poor perceptions or experiences of care and fear of providers and practices were common reasons to avoid certain health facilities, especially in the public sector. Use of public sector service was often considered a consequence of “helplessness” or when “in trouble”. Several studies affirm the urban preference for the private sector [35–37]. Among the reasons for this are ease of accessibility, convenient timings, and a perception that the quality of care is better than in the public sector [38–40]. However, access to private health facilities is limited by the ability to pay; some women who had particularly poor perceptions or experiences of public sector care had either sought financial support from within the family or had taken a loan to avoid seeking care at a public hospital. Muslim women were more likely to seek prenatal and delivery care at private hospitals, reflecting a strong preference for female physicians [15, 41].

Uptake of institutional care was lower among recent migrants to Mumbai. Of women who had arrived within the last year, 24 % made fewer than 3 prenatal visits and 39 % delivered at home. A study by Stephenson and Matthews [42] found that rural–urban migrant women in Mumbai reported levels of prenatal care similar to urban non-migrants but substantially lower delivery care, suggesting that migrants assimilated the urban preference for institutional prenatal care while preserving the traditional practice of home birth. One explanation was that while social networks provided women with information to access prenatal care, they were also a resource for home-based delivery care [42]. In a study of two migrant groups in a Delhi slum, institutional maternity care became habitual when modern health services were available and considered effective. Lower exposure to health care in the place of origin and unfamiliarity with hospital care resulted in greater fear and distrust of institutional delivery. Conversely, greater autonomy and social interaction outside the home increased women’s knowledge of health services and confidence to use them [12]. In our study, recent migrants had limited knowledge of health facilities and quality of services, and weak social networks. This reduces access to information about available or appropriate care and made it difficult to mobilise support to choose from a wider pool of providers. Women often seek maternity care from specific, local private providers recommended by family and reported to offer good quality care.

Our study contributes to an understanding of disparities in the utilisation of institutional care in poor urban areas by considering the complexity of factors that influence uptake and choice of provider across public and private sectors. Its strengths were a relatively large sample and disaggregated data on utilisation patterns in both public and private sectors. Limitations included potential recall bias and ‘best behaviour’ bias regarding women’s use of prenatal and delivery care. We have no reason to suspect that women gave false information, and the reported proportions of institutional care were similar to those in Mumbai slums as a whole [43]. Since we excluded families that were absent after the third visit, we might have missed some women who gave birth in their natal homes. A qualitative limitation arose from the use of quantitative and qualitative methods in grounded theory: we found it difficult to reconcile analytical concepts derived from deductive (quantitative) and inductive (qualitative) methods. We are continuing to develop our analysis into a substantive theory of provider selection.

Socioeconomic differentials manifest as inequities in the availability, affordability, and utilisation of health services [17, 44, 45]. The poorest are less able to pay for care because of disproportional health care costs from greater spending proportional to income, most of which have to be covered by wage income rather than savings [46]. Poorer groups, for whom good health and wellbeing are crucial for economic and household stability, often turn to more accessible, lower quality providers for their health care needs. They tend to consult with less competent practitioners who make less effort [47] or who operate in the largely-unregulated private sector. This is of concern because of the potential iatrogenic effects of over-medication, inappropriate treatment, or ignoring minimum standards of care [38, 48].

## Conclusions

In Mumbai’s informal settlements, institutional maternity care is the norm. Individuals and families, even in the most disadvantaged groups, choose among health providers in both private and public sectors. However, socio-economic inequalities limit people’s sphere of access and lead to differential utilisation across groups. Paradoxically, these inequalities make the selection of a suitable provider both more important and more difficult: more accessible practitioners are less likely to be fully qualified or trained, have lower competence and offer poorer quality care. Mitigating uncertainties about quality and safety compels many families to engage in a complex decision-making process, mediated by their ability to mobilise social and economic resources, in an attempt to ensure positive experiences and outcomes of care.

Addressing health care disparities in underserved communities requires a clear understanding of how families choose among health care options. In addition to questions of service uptake, research in pluralistic urban settings must disaggregate information by level of health facility and type of provider across sectors. Improving women’s choice and experiences of health care requires that health sector managers implement effective health system strategies, including high quality maternity services across sectors, a functioning regulatory mechanism, and monitoring of provider competences and behaviour.
